# Social determinants of health and all-cause or cardiovascular mortality on osteoarthritis adults in the USA: a national cohort study

**DOI:** 10.3389/fpubh.2025.1676418

**Published:** 2025-10-15

**Authors:** Ruliu Xiong, Xingmao Zhou

**Affiliations:** ^1^Zhongshan Hospital of Traditional Chinese Medicine Affiliated to Guangzhou University of Traditional Chinese Medicine, Zhongshan, Guangdong, China; ^2^Department of Orthopedics, Zhongshan Hospital of Traditional Chinese Medicine, Zhongshan, Guangdong, China

**Keywords:** osteoarthritis, social determinants of health, NHANES, mortality, cohort study

## Abstract

**Background:**

Social determinants of health (SDoH) are regarded as the fundamental causes of health and disease. Nevertheless, the relationship between SDoH and mortality risk in osteoarthritis (OA) patients remains poorly understood. This study aims to examine the associations between SDoH and all-cause or cardiovascular mortality risks among OA patients.

**Methods:**

Analysis of data from ten National Health and Nutrition Examination Survey (NHANES) cycles (1999–2018) encompassing 4,681 OA participants was conducted. Multivariable Cox proportional hazards models and Kaplan–Meier survival analyses were employed to assess the associations between SDoH and mortality outcomes, encompassing all-cause mortality and cardiovascular mortality. Restricted cubic spline (RCS) modeling was employed to assess potential non-linear associations. Subgroup analyses and interaction evaluations were subsequently performed to investigate the consistency of the observed associations across predefined demographic and clinical subgroups.

**Results:**

Over a median follow-up of 84 months, 1,300 participants died, including 447 cardiovascular deaths. In the fully adjusted multivariable model, Cox proportional hazards models showed that each 1-point increase in the cumulative SDoH score are associated with a 15% increased risk of all-cause mortality (HR = 1.15, 95% CI: 1.11–1.19) and a 13% elevated risk of cardiovascular mortality (HR = 1.13, 95% CI: 1.06–1.21). Most notably, Individuals with ≥5 adverse SDoH factors had a 119% higher risk of all-cause mortality (HR = 2.19, 95% CI: 1.72–2.79) and a 109% greater risk of cardiovascular mortality (HR = 2.09, 95% CI: 1.30–3.37) compared to those without any adverse factors. Kaplan–Meier survival curves further indicated significantly worse cumulative survival in high SDoH burden groups (Log-rank *p* < 0.001). Moreover, RCS analyses confirmed a linear dose–response gradient for SDoH levels and mortality risk (Non-linearity *p* > 0.05). Subgroup analyses identified stronger SDoH to all-cause mortality associations in low-BMI participants than high-BMI counterparts (Interaction *p* = 0.034).

**Conclusion:**

Among US adults with OA, adverse SDoH are associated with increased risks of all-cause mortality and cardiovascular mortality. Developing and implementing innovative public health approaches aimed at SDoH is crucial for mitigating premature mortality and addressing health inequities in this population. Integrating SDoH assessment into OA clinical management pathways and public health programs may improve prognostic outcomes; however, future research should validate these findings through large-scale prospective cohort studies and intervention trials.

## 1 Introduction

Osteoarthritis (OA), a highly prevalent musculoskeletal condition, stands as a primary driver of functional decline in the older population, constituting an escalating worldwide disease challenge affecting approximately 250 million individuals globally (1). Characterized as a degenerative joint disorder, OA manifests primarily through articular cartilage degradation, structural joint alterations, and joint inflammation, leading to pain and functional impairment (2). Per the Global Burden of Disease Study (2020), OA impacted 595 million people worldwide that year—equivalent to 7.6% of humanity (3). Driven by demographic aging and expanding populations, this high-burden condition is anticipated to increase in prevalence (4). Crucially, OA patients demonstrate excess mortality relative to the general population (5). A community-based longitudinal analysis of 2,156 subjects revealed OA’s systemic implications, showing significant links to both all-cause and cardiovascular disease (CVD) fatality (6). Moreover, pooled evidence indicates heightened CVD-specific mortality risk among OA patients, though its connection with all-cause death warrants further substantiation (7). These insights underscore the imperative to clarify OA-mortality relationships and pinpoint intervenable determinants to enhance outcomes and lower premature mortality.

The Healthy People 2030 framework advances health and well-being for all by guiding disease prevention and health promotion initiatives to reduce health disparities and advance health equity (8). The core objectives of Healthy People 2030 are intimately linked with the Social Determinants of Health (SDoH). The SDoH framework holistically evaluates conditions in the environments where people are born, live, learn, work, play, worship, and age (9). Mounting evidence demonstrates that SDoH exert pervasive impacts on health outcomes across all domains. Emerging studies demonstrate that adverse SDoH elevate premature mortality rates and underlie racial disparities in premature death from all causes among the U.S. population (10). Moreover, evidence indicates that adverse SDoH elevate risks of all-cause mortality and cause-specific mortality among people living with metabolic syndrome, rheumatoid arthritis, and chronic obstructive pulmonary disease (11–13). Even though SDoH are well-established contributors to the emergence and worsening of specific diseases, their connection to fatal outcomes in the OA population has received scant research attention. Most previous studies have focused on complex biological factors or single social factors, lacking systematic exploration of “upstream factors” like SDoH. Given the excessive and progressively increasing health burden and high mortality risk imposed by OA, pinpointing modifiable SDoH, creating specific public health approaches to boost healthcare access, and gauging health policy success in lessening disparities for OA patients are crucial.

Therefore, this study centrally aims to examine links between SDoH and death risk among OA patients, with particular focus on all-cause mortality and cardiovascular mortality in a nationally representative sample of U.S. adults. Our findings may yield new understanding about how “upstream” SDoH shape OA-associated mortality. Pinpointing modifiable SDoH factors tied to mortality could guide tailored public health programs and policies designed to alleviate inequities and curb preventable premature mortality in OA populations.

## 2 Materials and methods

### 2.1 Data source and study design

The National Health and Nutrition Examination Survey (NHANES), administered by the Centers for Disease Control and Prevention (CDC), represents a population-based surveillance program that monitors health and nutritional parameters in the United States. Employing age-stratified sampling methodologies, NHANES establishes a scientifically robust cross-sectional profile of U.S. population dynamics, enabling systematic surveillance of emergent disease patterns, modifiable health determinants, and population-level health trajectories. Through professional de-identification and unique identifier processing, the data were rendered suitable for public use. As the National Center for Health Statistics (NCHS) secured documented informed consent from all subjects, the current secondary data analysis was deemed exempt from further institutional ethics review.

### 2.2 Study population

**Figure 1:**
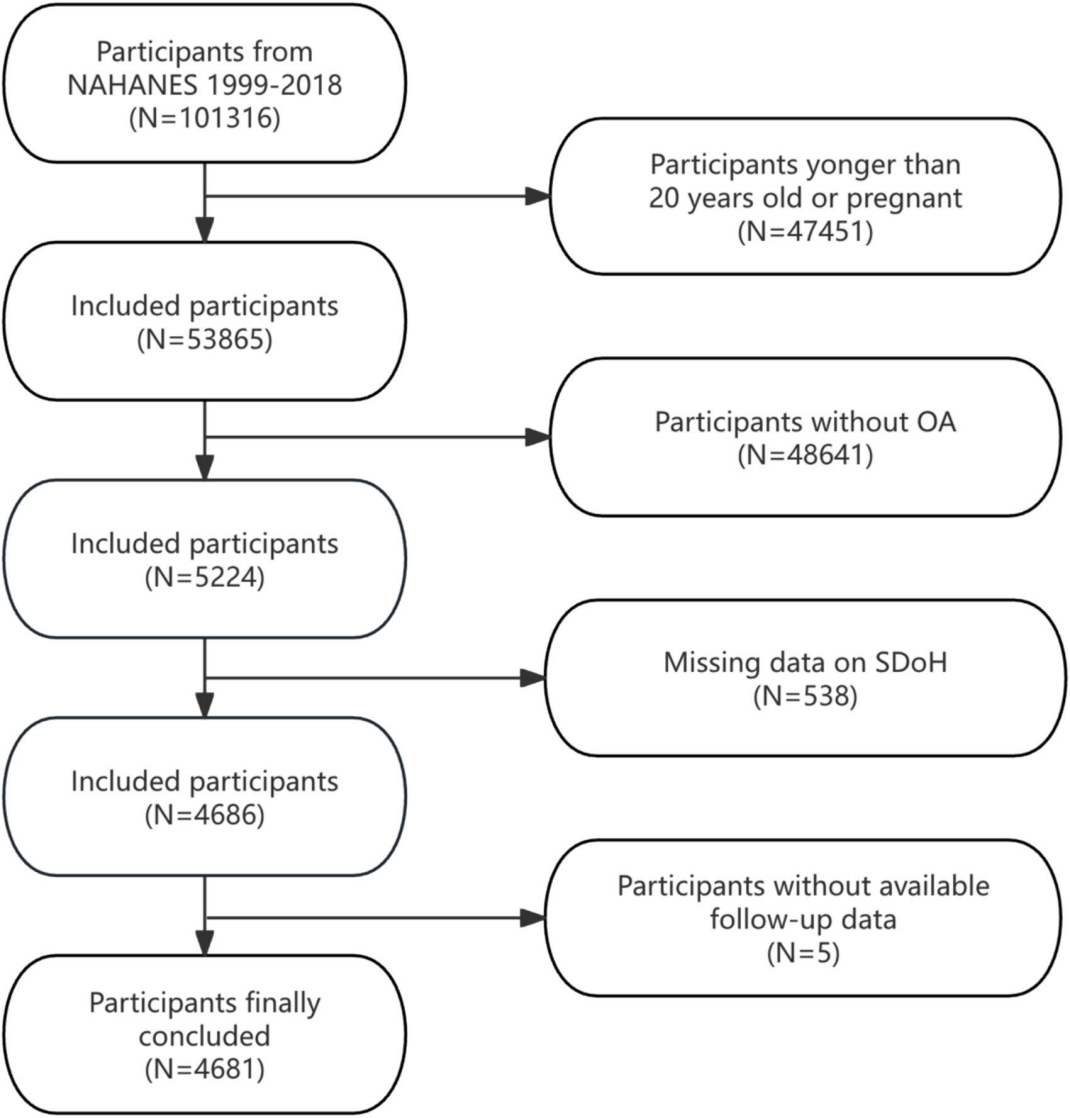
Flow diagram illustrating the enrollment process of participants in the present study.

This analysis aggregated data across 10 NHANES survey waves (1999–2000 to 2017–2018), initially including 101,316 individuals. To ensure sample comprehensiveness and relevance, we established specific inclusion and exclusion criteria. Exclusion criteria were as follows: (1) Participants younger than 20 years or pregnant (*n* = 47,451), (2) individuals not meeting OA diagnostic criteria (*n* = 48,641), (3) incomplete SDoH data (*n* = 538), and (4) lacking complete follow-up information (*n* = 5). After exclusions, the final analytical cohort comprised 4,681 eligible participants. Figure 1 details the selection process.

### 2.3 The definition of SDoH

Based on the Healthy People 2030 initiative and prior research, we assessed eight SDoH variables from NHANES data, categorized into the five SDoH domains (10). Each SDoH variable was dichotomized using conventional cut-points. To assess aggregated SDoH burden, we created a composite score by summing eight dichotomous SDoH measures, coded as 0 (favorable) or 1 (adverse). Due to limited observations with more than 5 adverse SDoH, values ≥5 were combined, yielding a final SDoH score range of 0 to ≥5. More detailed description of SDoH is provided in Supplementary Table S1.

### 2.4 Assessment of OA

OA diagnosis was derived from the NHANES Medical Conditions Questionnaire. This instrument initially screened participants with: “Has a doctor or other health professional ever told you that you have arthritis?” (yes/no). Affirmative responders were subsequently asked to specify arthritis type. Participants reporting “Osteoarthritis or degenerative arthritis” were defined as OA cases. Based on this questionnaire, individuals with rheumatoid arthritis, psoriatic arthritis, and other types of arthritis were also excluded. Due to the lack of radiological or clinical validation, this method cannot identify the specific anatomical location or subtype of osteoarthritis. Prior research demonstrates 81% concordance between self-reported OA and clinician-diagnosed OA, supporting the validity of self-reported data (14).

### 2.5 Mortality outcomes

The NCHS provided the public-use linked mortality files, with mortality outcomes determined through the National Death Index (NDI). Follow-up was conducted until December 31, 2019. The primary outcomes assessed were all-cause mortality and cardiovascular mortality (defined by ICD-10 codes: I00-I09, I11, I13, I20-I25, I26-I51, and I60-I69).

### 2.6 Covariates

Covariates included in the analysis spanned five domains: demographics (age, sex, race/ethnicity), anthropometric measures (body mass index [BMI], waist circumference), laboratory indicators (serum alanine aminotransferase [ALT] and aspartate aminotransferase [AST]), lifestyle factors (physical activity, drinking, smoking), and comorbidities (diabetes mellitus, hypertension, hyperlipidemia, cardiovascular disease [CVD]). Variable selection was guided by empirical evidence and theoretical frameworks derived from existing literature. Supplementary Table S2 provided detailed definitions and categorization methods.

### 2.7 Statistical analysis

All analyses incorporated sample weighting, stratification, and clustering to correct for oversampled subgroups, enhancing national representativeness. Specifically in accordance with NHANES recommendations, the wtint4yr variable was used to calculate sampling weights for each year within the two survey cycles spanning 1999–2002, while the wtint2yr variable was employed for the eight cycles spanning 2003–2018. Continuous variables with normal distribution are expressed as mean ± SD (standard deviation); between-group differences were assessed via weighted Student’s *t*-tests. Categorical variables, reported as counts and percentage, were compared using weighted χ^2^ tests.

Weighted Cox proportional hazards regression models were employed to examine the associations between SDoH and all-cause/cardiovascular mortality in OA patients. Outcomes are hazard ratios (HRs) with 95% CIs across three hierarchical models: Model 1: Crude (unadjusted). Model 2: Adjusted for demographic factors (age, sex, race/ethnicity). Model 3: Further adjusted for BMI, waist circumference, physical activity, drinking, smoking, diabetes, hypertension, hyperlipidemia, CVD, ALT, and AST. Restricted cubic spline (RCS) models were used to graphically explore the non-linear relationships between cumulative SDoH scores and all-cause mortality as well as cardiovascular mortality in patients with OA. Kaplan–Meier survival curves for all-cause and CVD survival were plotted, and survival status across different SDoH groups was compared using log-rank tests. Subgroup analyses examined effect modification by age, sex, race, BMI, drinking, smoking, physical activity, and health status (such as hypertension, diabetes, CVD, and hyperlipidemia). All computations used R software (version4.4.1) with survey package. Significance threshold: *p* < 0.05 (two-tailed).

## 3 Results

### 3.1 Participant characteristics

As shown in the Supplementary Table S3, the total weighted sample size included in the analysis comprised 20,149,092 individuals (unweighted *n* = 4,681). Baseline characteristics revealed that 65.4% were female and 34.6% male. A majority (58.4%) were under 65 years of age, and 59.0% identified as non-Hispanic White. Nearly half (46.0%) had a BMI ≥ 30 kg/m^2^. Prevalent health behaviors and conditions included drinking (73.5%), hypertension (62.7%), and hyperlipidemia (80.3%).

Significant differences were observed between survivors and decedents across multiple characteristics. Compared to survivors, decedents were significantly more likely to be older and of non-Hispanic White race/ethnicity. Decedents also exhibited a higher prevalence of hypertension, diabetes, and CVD, along with higher AST levels. Conversely, decedents had lower BMI, were more likely to be former smokers, reported lower physical activity levels, and were less likely to drinking. Marked disparities in social determinants were evident. Decedents were significantly more likely to have lower PIR, experience full food security, less than a high school education, lack a routine place for healthcare, be unmarried or not cohabiting with a partner and be less likely to hold private health insurance. Notably, the decedent group exhibited a significantly higher cumulative burden of adverse SDoH indicators.

### 3.2 Association between SDoH and mortality

Table 1 shows the outcomes of Cox proportional hazards regression models investigating the links between SDoH and all-cause mortality, with special attention to unfavorable SDoH continuous scores and categorical groupings. Elevated cumulative SDoH scores exhibited a significant, dose–response relationship with increased risks of all-cause and cardiovascular mortality among OA patients across progressively adjusted models (all *p* < 0.001). In the fully adjusted model (Model 3: age, sex, race, BMI, waist circumference, physical activity, drinking, smoking, diabetes, hypertension, hyperlipidemia, CVD, ALT, and AST), each 1-unit increase in the continuous SDoH score conferred a 15% higher risk of all-cause mortality (HR = 1.15, 95% CI: 1.11–1.191) and a 13% increased risk of cardiovascular mortality (HR = 1.13, 95% CI: 1.06–1.21). Categorical analysis demonstrated a pronounced gradient effect (P-trend<0.001), with mortality risks escalating progressively alongside accumulating adverse SDoH factors. Critically, compared to those with no adverse SDoH factors, patients with ≥5 adverse SDoH factors exhibited substantially elevated mortality risks: a 119% higher all-cause mortality risk (HR = 2.19, 95% CI: 1.72–2.79) and a 109% greater cardiovascular mortality risk (HR = 2.09, 95% CI: 1.30–3.37).

**Table 1:** Associations of social determinants of health and all-cause or cardiovascular mortality in participants with osteoarthritis from the NHANES 1999–2018.

SDoH variables	Model 1	*p*-value	Model 2	*p*-value	Model 3	*p*-value
	HR (95% CI)		HR (95% CI)		HR (95% CI)	
All-cause mortality						
SDoH, Continuous	1.18 (1.15–1.22)	<0.001	1.25 (1.21–1.30)	<0.001	1.15 (1.11–1.19)	<0.001
Cumulative SDoH variable						
0	1 (ref.)		1 (ref.)		1 (ref.)	
1	1.73 (1.36–2.21)	<0.001	1.44 (1.13–1.83)	0.003	1.23 (0.96–1.57)	0.108
2	2.44 (1.89–3.14)	<0.001	1.78 (1.40–2.26)	<0.001	1.42 (1.11–1.83)	0.006
3	3.14 (2.39–4.14)	<0.001	2.39 (1.83–3.12)	<0.001	1.78 (1.37–2.32)	<0.001
4	3.26 (2.51–4.23)	<0.001	2.88 (2.19–3.80)	<0.001	1.82 (1.39–2.37)	<0.001
≥5	2.67 (2.13–3.35)	<0.001	3.56 (2.80–4.52)	<0.001	2.19 (1.72–2.79)	<0.001
P for trend		<0.001		<0.001		<0.001
Cardiovascular mortality						
SDoH, Continuous	1.19 (1.13–1.26)	<0.001	1.28 (1.20–1.36)	<0.001	1.13 (1.06–1.21)	<0.001
Cumulative SDoH variable						
0	1 (ref.)		1 (ref.)		1 (ref.)	
1	1.80 (1.10–2.93)	0.018	1.54 (0.95–2.49)	0.077	1.26 (0.77–2.05)	0.357
2	3.07 (1.93–4.89)	<0.001	2.34 (1.49–3.69)	<0.001	1.72 (1.08–2.74)	0.023
3	4.16 (2.51–6.88)	<0.001	3.45 (2.09–5.69)	<0.001	2.27 (1.36–3.80)	0.002
4	3.25 (1.96–5.38)	<0.001	3.08 (1.83–5.21)	<0.001	1.63 (0.97–2.71)	0.063
≥5	2.84 (1.69–4.76)	<0.001	4.02 (2.41–6.71)	<0.001	2.09 (1.30–3.37)	0.002
P for trend		<0.001		<0.001		<0.001

Model 1: no covariates were adjusted. Model 2: age, sex, and race were adjusted. Model 3: age, sex, race, BMI, waist circumference, physical activity, drinking, smoking, diabetes, hypertension, hyperlipidemia, CVD, ALT, and AST. Abbreviation: SDoH, Social determinants of health; HR, hazard ratio; CI, confidence interval.

### 3.3 Associations between individual SDOH component and mortality

After fully adjusting for all covariates in Model 3, we analyzed the associations between individual components of SDoH and all-cause mortality or cardiovascular mortality. As shown in Table 2, five adverse SDoH factors significantly increased all-cause mortality risk: unemployed (HR = 1.56, 95% CI:1.28–1.90), poverty-income ratio <3 (HR = 1.41, 1.20–1.64), less than high school (HR = 1.42, 1.24–1.62), rent or other arrangements (HR = 1.24, 1.06–1.45), and not married or no partner (HR = 1.37, 1.19–1.57). Conversely, for cardiovascular mortality, only four factors showed significant associations: unemployed (HR = 1.67, 1.26–2.22), poverty-income ratio <3 (HR = 1.56, 1.19–2.04), less than high school (HR = 1.68, 1.33–2.13), and marital status (HR = 1.37, 1.07–1.75).

**Table 2:** Associations of individual components of social determinants of health and all-cause or cardiovascular mortality in participants with osteoarthritis from the NHANES 1999–2018.

Individual components of SDoH	All-cause mortality		Cardiovascular mortality	
	HR (95% CI)	*p*-value	HR (95% CI)	*p*-value
Employment (unemployed vs. employed)	1.56 (1.28–1.90)	<0.001	1.67 (1.26–2.22)	<0.001
Poverty-income ratio (<3vs ≥ 3)	1.41 (1.20–1.64)	<0.001	1.56 (1.19–2.04)	0.001
Food security (insecure vs. secure)	1.16 (0.95–1.43)	0.148	0.90 (0.63–1.28)	0.545
Education (less than high school vs. high school or more)	1.42 (1.24–1.62)	<0.001	1.68 (1.33–2.13)	<0.001
Healthcare (no routine place vs. routine place to go)	0.84 (0.59–1.22)	0.363	0.44 (0.17–1.13)	0.089
Health insurance (government or no insurance vs. private insurance)	1.12 (0.97–1.30)	0.126	1.14 (0.90–1.45)	0.287
Housing instability (rent or other arrangements vs. own a home)	1.24 (1.06–1.45)	0.009	1.05 (0.79–1.38)	0.754
Marital status (not married or no partner vs. married or partnered)	1.37 (1.19–1.57)	<0.001	1.37 (1.07–1.75)	0.012

The HR was adjusted for participant’s age, sex, race, BMI, waist circumference, physical activity, drinking, smoking, diabetes, hypertension, hyperlipidemia, CVD, ALT, and AST. CI, confidence interval; HR, hazard ratio; SDoH, Social determinants of health.

### 3.4 Kaplan–Meier survival curves

**Figure 2:**
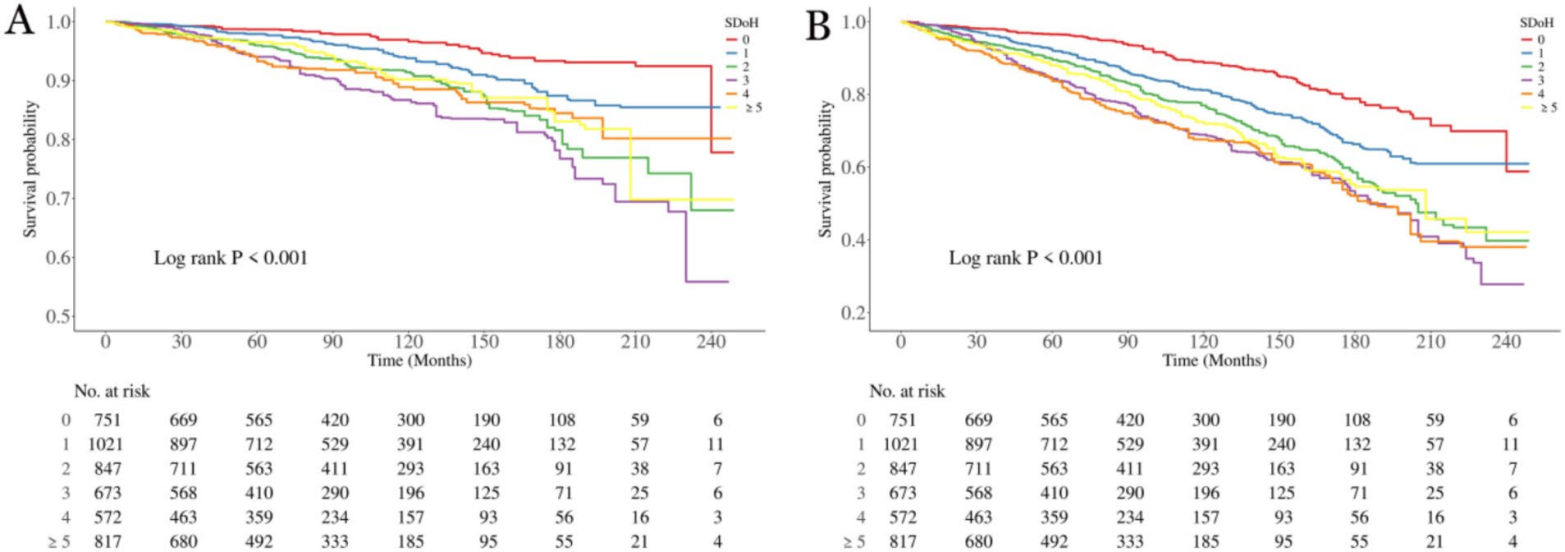
Kaplan–Meier analyses for mortality among the SDoH groups. **(A)** All-cause mortality; **(B)** Cardiovascular mortality. SDoH, social determinants of health.

To elucidate the associations between SDoH and all-cause mortality as well as cardiovascular mortality in patients with OA, we employed the Kaplan–Meier analyses (Figure 2). A total of 1,300 participants died during a median follow-up period of 84 months (interquartile range: 46–137 months), among whom 447 deaths were attributed to CVD. The results showed that the survival probabilities of all-cause mortality and cardiovascular disease were significantly associated with the level of SDOH (*p* < 0.001 for all log-rank tests).

### 3.5 RCS analysis

**Figure 3:**
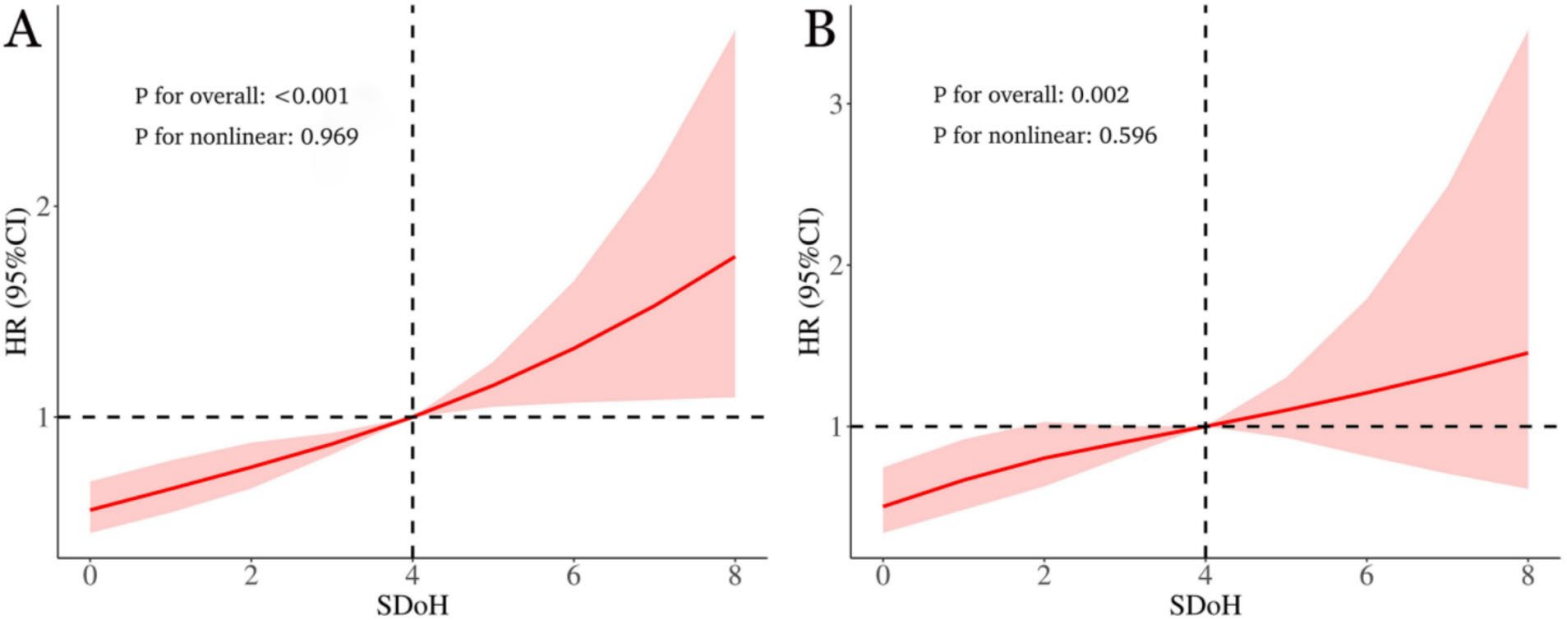
The link between social determinants of health (SDoH) and all-cause **(A)** as well as cardiovascular mortality **(B)** in osteoarthritis patients is illustrated via restricted cubic spline. The hazard ratios (solid lines) and 95% confidence intervals (shaded areas) were adjusted for age, sex, race, BMI, waist circumference, physical activity, drinking, smoking, diabetes, hypertension, hyperlipidemia, CVD, ALT, and AST.

As shown in Figure 3, within the fully adjusted RCS model that controlled for full confounding variables, a higher SDoH score among OA patients correlated with elevated risks of all-cause and cardiovascular mortality. Moreover, this association exhibited a linear relationship (*p*-values for non-linearity were 0.969 and 0.596, respectively).

### 3.6 Subgroup analysis

**Figure 4:**
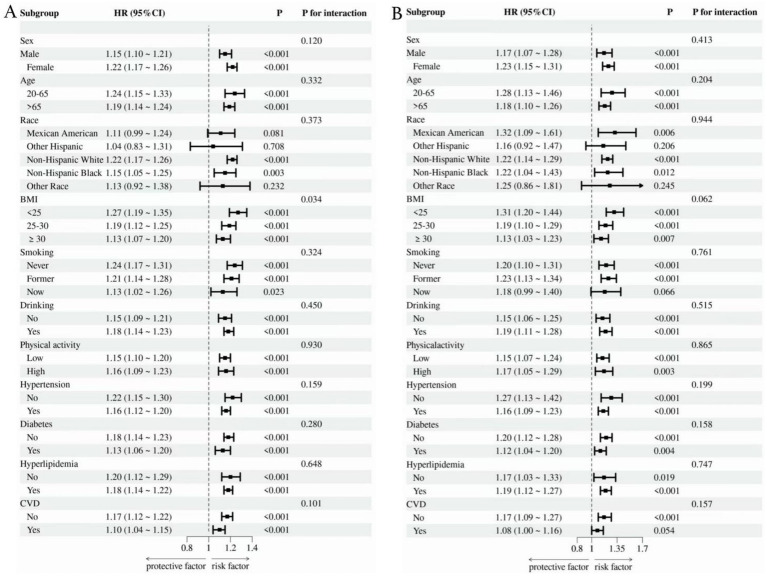
Subgroup analysis of the link between SDoH and all-cause **(A)** as well as cardiovascular mortality **(B)**.

As shown in Figure 4, we conducted subgroup analyses of OA patients according to factors including gender, age, race, BMI, smoking, drinking, physical activity, diabetes, hypertension, hyperlipidemia, and CVD. The findings indicated that SDoH was positively correlated with the risks of all-cause mortality and cardiovascular mortality. Furthermore, the interaction between BMI and SDoH exerted a significant effect on all-cause mortality (interaction *p*-value = 0.034), with individuals having a lower BMI being more prone to an elevated risk of all-cause mortality compared to those with a higher BMI. Importantly, no significant interactions were identified in other subgroups, suggesting that the influence of SDoH on all-cause mortality and cardiovascular mortality in OA patients remains consistent across different subgroups.

## 4 Discussion

This study is the first to investigate the links between SDoH and mortality outcomes (including all-cause mortality and cardiovascular mortality) in OA patients utilizing NHANES data. A total of 4,681 OA participants were included between 1999 and 2018, with 1,300 deaths occurring during the follow-up period—447 of which were cardiovascular deaths. There are 3 main findings. First, after comprehensive adjustment for confounding variables, results indicated that a higher SDoH score was significantly and linearly correlated with elevated risks of all-cause and cardiovascular mortality in OA patients. Second, an analysis of the eight individual SDoH components revealed that employment status, PIR, educational attainment, and marital status were collectively correlated with both all-cause and cardiovascular mortality to varying degrees, while housing instability was independently associated with an elevated risk of all-cause mortality. Third, interaction analyses revealed that OA patients with a lower BMI might face a higher risk of all-cause mortality compared to those with a higher BMI. This study offers new epidemiological evidence connecting SDoH to mortality risk in OA, laying a foundation for the equitable implementation of health policies aimed at improving prognosis and reducing excess deaths in this vulnerable population.

Osteoarthritis ranks among the most disabling conditions globally, significantly undermining physical function and quality of life (15). As populations age, the epidemiological load of osteoarthritis is projected to grow. It is worth emphasizing that individuals with OA have higher mortality rates than the general population (5, 16, 17). The underlying mechanisms driving this excess mortality remain incompletely understood (18). Substantial research activity has recently emerged to address this knowledge gap, investigating diverse factors including biomarkers, dietary intake patterns, pharmacological exposures, anthropometric measurements, and comorbidity profiles (19–29). However, a pivotal challenge in OA management is the continued absence of disease-modifying osteoarthritis drugs (DMOADs), which restricts therapeutic options to symptomatic pain management and lifestyle adaptations (30, 31). Consequently, OA prognosis is fundamentally contingent upon socioeconomic mediators, healthcare accessibility, and integrated multidisciplinary interventions targeting biomechanical, metabolic, and psychosocial determinants of health (32).

Efforts in research and practice aimed at advancing health equity have a long-standing history. The 1986 Ottawa Charter was the first to explicitly coin the term “social determinants of health” and stressed that “health should be attained through social policies.” In 2008, the report issued by the World Health Organization (WHO) pointed out that the root cause of health inequalities lies in social injustice rather than biomedical differences, thereby raising attention to SDoH to a new level. In recent years, an increasing body of research has examined the link between SDoH and adverse clinical outcomes, such as recurrent preschool wheeze (33), intellectual disability (34), hearing loss (35), awareness of diabetic retinopathy (36), premature death (10), and so on. Like the work by Bundy et al., our study focuses on the link between SDoH and mortality. It is important to note that the effect of SDoH on the risk of death differs across various populations (37), and our study population consists of OA patients, which is what distinguishes this study. Similarly, within the OA population, a significant impact of SDoH on mortality risk was also confirmed. In this study, we comprehensively accounted for potential confounding factors, including participant’s age, sex, race, BMI, waist circumference, physical activity, drinking, smoking, diabetes, hypertension, hyperlipidemia, CVD, ALT, and AST, demonstrating the robustness of the results. We utilized Cox proportional hazards regression models and RCS to analyze the relationship between SDOH and mortality, revealing a linear association. Kaplan–Meier survival curves further corroborated this association. Subgroup and interaction analyses validated the consistency of these findings. Additionally, we individually examined the relationship between each SDOH component and mortality, identifying the primary drivers underlying this association. These findings provide valuable insights for developing targeted interventions.

As a key component of the SDoH hierarchical model, economic stability is a fundamental dimension influencing health outcomes. Both WHO and Commission on Social Determinants of Health (CSDH)classify it as a foundational level of SDoH, given its role as a prerequisite for accessing other health resources. In our study, economic stability includes employment status, PIR, and food security. It was found that both employment status and the poverty-income ratio (PIR) are significantly linked to the risk of death. Specifically, unemployment was correlated with a 56% higher risk of all-cause mortality and a 67% higher risk of cardiovascular mortality, while a PIR below 3 was associated with a 41% higher risk of all-cause mortality and a 56% higher risk of cardiovascular mortality. In comparison, socioeconomic status (SES), which has been widely utilized in prior research, has also been demonstrated to correlate with various health-related outcomes. Besides income and occupation, education constitutes another key component of SES. The influence of SES on OA patients is well-established (38). For example, in the Johnston County Osteoarthritis Project, a notable connection was observed between low SES and adverse disability/pain outcomes in older adults with hip radiographic osteoarthritis (rOA) (39). Likewise, another study indicated that both lower individual and community SES correlate with poorer function and pain in adults with knee rOA (40). A Swiss study involving 166,076 patients with primary osteoarthritis revealed that those facing socioeconomic disadvantages have an elevated risk of early mortality and morbidity following total hip replacement (41). These results suggest that socioeconomic status disadvantages (such as lack of economic resources and insufficient educational opportunities) may indirectly increase the risk of death in OA patients through pathways such as limiting healthcare accessibility, exacerbating health behavior risks (e.g., insufficient physical activity, smoking), or causing long-term psychological stress (42). Individuals with low educational attainment may struggle to understand disease management knowledge, leading to poor adherence. For them, even if they receive self-management advice, the idea that exercise is safe for their joints may still seem counterintuitive (43). Unemployed individuals may be unable to afford long-term treatment costs due to unstable income, which further exacerbates their condition. However, another study observed that patients with osteoarthritis who are better managed consistently have higher SES (38). In contrast, vulnerable groups, despite bearing a heavier disease burden, struggle to benefit from existing management programs due to limited access to resources. This discrepancy highlights the limitations of current OA interventions in terms of social structural equity: standardized self-management programs may exacerbate health inequalities by failing to adapt to SES differences. To achieve the goal of Universal Health Coverage (UHC), it is necessary to integrate SES stratification strategies into the design of healthcare systems.

In our study, marital status emerged as a critical socioeconomic factor influencing mortality risk among OA patients. Specifically, unpartnered individuals (unmarried or non-cohabiting) exhibited 37% higher hazards of all-cause and cardiovascular mortality. As a fundamental lifelong social bond, marriage profoundly impacts health through psychological/economic support and behavioral regulation. A major prospective Chinese cohort revealed 29 and 4% excess mortality among unpartnered men and women, respectively, versus partnered counterparts (44). Additionally, married patients demonstrated superior functional recovery post-total knee arthroplasty (45). The mortality risk associated with solitary living may stem from reduced social scaffolding. Successful marital partnerships foster emotional resilience and mutual psychological well-being. Moreover, stable marriages enhance pain-coping capacities, mitigating activity limitations (46). Concurrently, housing instability was independently associated with elevated all-cause mortality in our cohort. Residential precarity represents a mounting public health challenge in industrialized societies, especially across the U.S. Research on its health consequences remains limited. Existing evidence implicates multifactorial pathways: housing hardship compromises psychological health by amplifying stress and depressive symptoms, potentiating high-risk behaviors (e.g., smoking, heavy drinking, cannabis use) (47–49). It also reflects underlying financial toxicity, forcing trade-offs between housing costs and medications (50) while impeding treatment adherence and disease management (51). Critically, in our study, renters and those with other arrangement were associated with a 24% higher mortality risk, highlighting that housing security is a pivotal intervention target for reducing health inequities and premature death. Thus, OA care frameworks must prioritize patients experiencing housing adversity—a population with demonstrated vulnerability to poor outcomes.

Furthermore, in our study, we observed a paradoxical relationship between BMI and mortality among OA patients. Participant characteristics indicated that nearly half (46.0%) had a BMI ≥ 30 kg/m^2^, consistent with extensive evidence linking obesity to increased OA risk. Notably, our interaction analysis revealed that the BMI-SDoH interaction significantly impacted all-cause mortality. Counter to expectations, reduced BMI predicted higher mortality risk relative to elevated BMI—a phenomenon observed in other pathologies (52, 53). For instance, pooled evidence indicates subnormal BMI elevates post-stroke mortality, potentially reflecting adipose tissue’s protective functions (52). It should be noted that our study exclusively enrolled a population with osteoarthritis, with a mean age of 61.66 ± 13.29 years. Non-survivors were notably older (mean age 71.17 ± 10.64 years), suggesting that the overall cohort is relatively advanced in age. A prospective cohort study involving 166,285 Chinese adults similarly observed a protective effect of higher adiposity in older individuals—increased all-cause mortality among underweight participants may be attributable to reduced lean body mass (54). Previous studies have indicated that lower lean mass or fat-free mass in low-BMI individuals is significantly associated with an elevated risk of all-cause mortality, underscoring the importance of distinguishing between healthy and unhealthy leanness when interpreting excess mortality in underweight populations (55–57). As a crucial metabolic organ, loss of muscle mass is strongly linked to frailty, falls, impaired immune function, and higher mortality (58). This risk is particularly pronounced in patients with osteoarthritis due to joint pain and limited mobility (59). Another large-scale cohort study of 3.6 million adults in the UK showed that all-cause mortality among older adults reached its lowest point at higher BMI levels, indicating that nutritional reserves may play an increasingly important role in this age group (60). On one hand, low body weight related to malnutrition may weaken immune function and increase susceptibility to infections. On the other hand, reverse causality is more likely in older populations due to a higher prevalence of comorbidities—preexisting chronic conditions can lead to both weight loss and an increased mortality risk. Collectively, these findings argue for a more nuanced approach to weight management in older adults, suggesting that public health guidelines and clinical recommendations should move beyond BMI-based thresholds and incorporate age-specific considerations, as well as more precise indicators of body composition such as Body Roundness Index, Weight-adjusted Waist Index, or direct measures of lean mass and fat distribution.

It should be acknowledged that several limitations of this study should be considered when interpreting the results. First, despite mortality data linkage with the NDI, NHANES’ cross-sectional design precludes causal inference. Second, although multivariable models incorporated extensive covariates, residual confounding may limit result generalizability and precision. In addition, due to the breadth and richness of SDoH, our selection of them is biased toward some relatively important and common factors, and there are still some significant SDoH not included in our investigation, such as experiences of racism, discrimination, and social support. Thus, external validation cohorts and mechanistic investigations through large-scale prospective studies and interventional trials are imperative. Moreover, the NHANES database lacks detailed economic measures—such as out-of-pocket medical costs or cost-related non-adherence—which limited our ability to examine financial aspects of SDoH in relation to mortality, as noted by a reviewer. Future studies incorporating economic burden are warranted to extend this important direction. While findings primarily reflect U.S. populations, extrapolation to other ethnic/geographic groups requires caution.

## 5 Conclusion

Within a nationally representative sample of U.S. adults, adverse SDoH were strongly associated with an excess risk of all-cause mortality and cardiovascular disease mortality in patients with OA patients. Critical determinants included employment status, household income, educational attainment, residential instability, and marital status—all independently associated with fatal outcomes in OA patients. Integrating SDoH assessment into OA clinical management protocols and public health initiatives may enhance prognostic outcomes, though future research should validate these findings through large-scale prospective cohorts and interventional trials.

## Data Availability

Publicly available datasets were analyzed in this study. This data can be found here: the survey data are publicly accessible and can be freely downloaded from the NHANES website (https://wwwn.cdc.gov/nchs/nhanes/default.aspx) by users and researchers around the globe. No additional datasets were generated or analyzed during the current study.
