# Supplementary material for: Social determinants of health and all-cause or cardiovascular mortality on osteoarthritis adults in the USA: a national cohort study
Source: Front Public Health. 2025 Oct 15;13:1676418. doi: 10.3389/fpubh.2025.1676418 (PMC12568563; doi:10.3389/fpubh.2025.1676418)
Supplement: Supplementary file 1 [file Table_1.docx]

Supplementary Material

**Table S1**. Definitions of social determinants of health domains and sub-items.

| **Domain** | **Sub-items** | **Categories** | **Definitions** |
| --- | --- | --- | --- |
| **Economic stability** | **Employment** | 0: Employed, student, or retired  1: Unemployed | Participants were asked what type of work they have done in the last week and, if not working, the main reason why. All participants not working were classified as unemployed, except those who responded they were a student or retired were grouped with those reporting employment. |
|  | **Poverty/ income ratio** | 0: ≥300%  1: <300% | Participants were asked their family income and size, and the poverty/income ratio is the ratio of family income to poverty. The Department of Health and Human Services poverty guidelines were used as the poverty measure to calculate this ratio. These guidelines are issued each year, in the Federal Register, for determining financial eligibility for certain federal programs. The guidelines vary by family size and geographic location. |
|  | **Food security** | 0: Full security  1: Marginal, low, or very low security | Participants responded to the U.S. Food Security Survey Module questions whether: 1) they were worried if food would run out before there was money to buy more; 2) the food they bought didn’t last and they didn’t have money to get more; 3) they couldn’t afford to eat balanced meals; 4) they had cut the size of meals or skipped meals because there wasn’t enough money for food; 5) if yes to #4, how often meals were cut or skipped; 6) they ate less than they felt they should because there was not enough money to buy food; 7) they were hungry but didn’t eat because they couldn’t afford food; 8) they lost weight because they didn’t have enough money for food; 9) they did not eat for a whole day because there was not enough money for food; 10) if yes to #9, how often they did not eat for the whole day. Levels of food security were classified as follows: full food security, no affirmative responses; marginal food security, 1-2 affirmative responses; low food security, 3-5 affirmative responses; and very low food security, 6-10 affirmative responses. |
| **Education access and quality** | **Education level** | 0: High school or more  1: Less than high school | Participants were asked for the highest grade or level of school they completed. The response categories are: less than 9th grade education, 9-11th grade education (includes 12th grade and no diploma), High school graduate/GED, some college or associates (AA) degree, and college graduate or higher. |
| **Healthcare access and quality** | **Access to healthcare** | 0: Yes  1: No | Participants were asked if there is a place they usually go when sick or needing advice about health. If they answered “yes” or “there is more than one place” to this question, they were classified as having a routine place for healthcare. If yes, but the facility is a hospital emergency room, they were classified as not having a routine place for healthcare. |
|  | **Health insurance** | 0: Private insurance  1: Government or no insurance | Participants were asked whether they are covered by health insurance or some other kind of health care plan. They are subsequently asked if covered by private insurance or several types of government insurance (Medicare, Medi-Gap, Medicaid, SCHIP, military health care, Indian Health Service, state-sponsored health plan, or other government insurance). |
| **Neighborhood and built environment** | **Housing instability** | 0: Own home  1: Rent home or other arrangement | Participants were asked if the home they are living in is owned, being bought, rented, or occupied by some other arrangement. A person was considered to own the home even if they are still paying on a mortgage. |
| **Social and community context** | **Marital status** | 0: Married or living with a partner  1: Not married or living with a partner | Participants were asked whether they were married, widowed, divorced, separated, never married, or living with a partner. Those reporting marriage or living with a partner were grouped together. |

**Table S2**. Definition of variables involved in this study.

|  | **Covariates** | **Description in NHANES** |
| --- | --- | --- |
| **Demographics** | **Age** | Divided into two groups: 20-65 years old, >65 years old |
|  | **Sex** | Male and Female |
|  | **Race** | Mexican American, Other Hispanic, Non-Hispanic Black, Non-Hispanic White, Other Race |
| **Anthropometric measures** | **BMI** | BMI is calculated as weight (in kilograms) divided by the square of height (in meters). BMI is classified into three groups: < 25 kg/m², 25 - 30 kg/m², and ≥ 30 kg/m². |
|  | **Waist circumference** | Waist circumference (centimeter) as a continuous variable. |
| **Laboratory indicators** | **ALT** | Alanine aminotransferase(U/L). |
|  | **AST** | Aspartate aminotransferase(U/L). |
| **Lifestyle** | **Smoking** | Smoking was grouped into 3 categories: was categorized as never (lifetime consumption <100 cigarettes), former (>100 cigarettes with cessation), and current (>100 cigarettes with continued use) |
|  | **Drinking** | Drinking was classified as “yes” or “no” by whether≥4 drinks/day |
|  | **Physical activity** | For physical activities, calculate the Metabolic Equivalent of Task (MET) score of a specific activity based on its type and intensity. Participants were classified into two categories: low physical activity (<500 MET-minutes/week) and high physical activity (≥500 MET-minutes/week) |
| **Comorbidities** | **Diabetes** | Diabetes was defined as a history of previous diabetes, hypoglycemic medication use, or meeting anyone biochemical thresholds: hemoglobin A1c (HbA1c) ≥6.5% or fasting blood glucose ≥126 mg/dL or postprandial 2-hour blood glucose ≥200mg/dL |
|  | **Hypertension** | The diagnostic criteria consist of self-reported hypertension history, the utilization of antihypertensive medication, an average systolic blood pressure (SBP) ≥ 140mmHg, or an average diastolic blood pressure (DBP) ≥ 90mmHg |
|  | **Hyperlipidemia** | Hyperlipidemia was defined as the use of lipid-lowering medications or meeting any of the following criteria: total cholesterol ≥ 200 mg/dL or triglycerides≥150 mg/dL or low-density lipoprotein cholesterol levels≥ 130 mg/dL or gender-specific high-density lipoprotein cholesterol thresholds (males ≤40 mg/dL, females ≤50 mg/dL) |
|  | **CVD** | CVD history is obtained from the Medical Conditions Questionnaire and defined as those with stroke, congestive heart failure, coronary heart disease, heart attack, myocardial infarction, or angina pectoris. |

CVD, cardiovascular disease.

**Table S3** The basic characteristics of the osteoarthritis population from the NHANES 1999–2018 were stratified by survival status (weighted).

| **Characteristic** | **Overall,**  **N=20149092** | **Survivor**  **N=16002940** | **Non-survivors**  **N=4146152** | ***p-value*** |
| --- | --- | --- | --- | --- |
| No. of participants in the sample | 4681 | 3381 | 1300 |  |
| Age (mean (SD)) | 61.66 (13.29) | 59.20 (12.79) | 71.17 (10.64) | <0.001 |
| Age (%) |  |  |  | <0.001 |
| 20-65 | 11762271 (58.4) | 10639197(66.5) | 1123074(27.1) |  |
| >65 | 8386820 (41.6) | 5363742 (33.5) | 3023078(72.9) |  |
| Sex (%) |  |  |  | 0.224 |
| Male | 6980628 (34.6) | 5463257 (34.1) | 1517371 (36.6) |  |
| Female | 13168464 (65.4) | 10539683 (65.9) | 2628781 (63.4) |  |
| Race (%) |  |  |  | <0.001 |
| Mexican American | 551763 (2.7) | 487286(3.0) | 64477(1.6) |  |
| Other Hispanic | 467306(2.3) | 389768(2.4) | 77538(1.9) |  |
| Non-Hispanic White | 16870370(83.7) | 13219065(82.6) | 3651305 (88.1) |  |
| Non-Hispanic Black | 1252542 (6.2) | 1032776(6.5) | 219766 (5.3) |  |
| Other Race | 1007112(5.0) | 874046 (5.5) | 133066 (3.2) |  |
| BMI (%) |  |  |  | <0.001 |
| <25 | 4310185(21.4) | 3254940(20.3) | 1055245 (25.5) |  |
| 25-30 | 6563083 (32.6) | 5093798 (31.8) | 1469285 (35.4) |  |
| ≥30 | 9275824(46.0) | 7654202 (47.8) | 1621622(39.1) |  |
| Waist circumference (mean (SD)) | 103.89 (16.52) | 104.06 (16.44) | 103.25 (16.81) | 0.244 |
| Smoking (%) |  |  |  | 0.003 |
| Never | 9559922 (47.4) | 7801223 (48.7) | 1758699(42.4) |  |
| Former | 7353855 (36.5) | 5591976(34.9) | 1761879 (42.5) |  |
| Now | 3235316(16.1) | 2609740(16.3) | 625575(15.1) |  |
| Drinking (%) |  |  |  | <0.001 |
| No | 5348176 (26.5) | 3815299 (23.8) | 1532877(37.0) |  |
| Yes | 14800916(73.5) | 12187641(76.2) | 2613275 (63.0) |  |
| Physical activity (%) |  |  |  | <0.001 |
| Low | 9028367 (44.8) | 6582057 (41.1) | 2446310 (59.0) |  |
| High | 11120725(55.2) | 9420883(58.9) | 1699842 (41.0) |  |
| Hypertension (%) |  |  |  | <0.001 |
| No | 7511653 (37.3) | 6600701 (41.2) | 910952(22.0) |  |
| Yes | 12637439 (62.7) | 9402239(58.8) | 3235200(78.0) |  |
| Diabetes (%) |  |  |  |  |
| No | 15673497 (77.8) | 12756441(79.7) | 2917056 (70.4) | <0.001 |
| Yes | 4475595(22.2) | 3246498 (20.3) | 1229097 (29.6) |  |
| Hyperlipidemia (%) |  |  |  |  |
| No | 3960599 (19.7) | 3208507(20.0) | 752092(18.1) | 0.193 |
| Yes | 16188493(80.3) | 12794433(80.0) | 3394060(81.9) |  |
| CVD (%) |  |  |  |  |
| No | 16196348 (80.4) | 13569379 (84.8) | 2626969(63.4) | <0.001 |
| Yes | 3952744(19.6) | 2433561(15.2) | 1519183(36.6) |  |
| ALT (mean (SD)) | 23.55 (14.28) | 23.79 (13.85) | 22.66 (15.80) | 0.111 |
| AST (mean (SD)) | 25.11 (13.44) | 24.51 (10.69) | 27.42 (20.75) | 0.004 |
| SDoH score (mean (SD)) | 1.95 (1.78) | 1.83 (1.78) | 2.40 (1.72) | <0.001 |
| SDoH (%) |  |  |  | <0.001 |
| 0 | 4868374 (24.2) | 4353475(27.2) | 514899(12.4) |  |
| 1 | 5273644(26.2) | 4296163(26.8) | 977481 (23.6) |  |
| 2 | 3616559(17.9) | 2720790(17.0) | 895769 (21.6) |  |
| 3 | 2273314(11.3) | 1580490(9.9) | 692824 (16.7) |  |
| 4 | 1841981 (9.1) | 1298794(8.1) | 543187 (13.1) |  |
| ≥5 | 2275219 (11.3) | 1753227 (11.0) | 521992 (12.6) |  |
| Employment status (%) |  |  |  | 0.157 |
| Employed, student, or retired | 16040640 (79.6) | 12824109 (80.1) | 3216531 (77.6) |  |
| Not employed | 4108453(20.4) | 3178831 (19.9) | 929622 (22.4) |  |
| Poverty-income ratio (%) |  |  |  | <0.001 |
| ≥ 3 | 10669140 (53.0) | 9127147 (57.0) | 1541993 (37.2) |  |
| <3 | 9479952(47.0) | 6875793 (43.0) | 2604159(62.8) |  |
| Food security (%) |  |  |  | 0.004 |
| Full food security | 16676113(82.8) | 13101400 (81.9) | 3574713 (86.2) |  |
| Marginal, low, or very low | 3472979 (17.2) | 2901539(18.1) | 571440(13.8) |  |
| Education level (%) |  |  |  | <0.001 |
| High school or more | 17233643 (85.5) | 14159639(88.5) | 3074004(74.1) |  |
| Less than high school | 2915448(14.5) | 1843300(11.5) | 1072148 (25.9) |  |
| Healthcare (%) |  |  |  | <0.001 |
| Routine place to go for healthcare | 19110809 (94.8) | 15073771(94.2) | 4037038(97.4) |  |
| No routine place, or ER/hospital/other | 1038283(5.2) | 929169(5.8) | 109114(2.6) |  |
| Health insurance (%) |  |  |  | <0.001 |
| Private insurance | 12929763(64.2) | 10619105(66.4) | 2310658 (55.7) |  |
| Government or no insurance | 7219328 (35.8) | 5383834(33.6) | 1835494(44.3) |  |
| Home ownership (%) |  |  |  | 0.049 |
| Own home | 16420869 (81.5) | 13154995 (82.2) | 3265874(78.8) |  |
| Rent or other arrangement | 3728223(18.5) | 2847945(17.8) | 880278(21.2) |  |
| Marital status (%) |  |  |  | <0.001 |
| Married or living with a partner | 12823025 (63.6) | 10618243(66.4) | 2204782 (53.2) |  |
| Not married nor living with a partner | 7326067 (36.4) | 5384697 (33.6) | 1941370 (46.8) |  |

Continuous variables: Expressed as mean ± standard deviation; group differences assessed via weighted Student's t-tests. Categorical variables: Reported as weighted frequencies (N, %); associations evaluated using weighted χ² tests. Abbreviations: SDoH, Social determinants of health; PIR, poverty income ratio; ALT, alanine aminotransferase; AST, aspartate aminotransferase; CVD, cardiovascular disease.
